# Supplementary material for: c-Abl Tyrosine Kinase Is Required for BDNF-Induced Dendritic Branching and Growth
Source: Int J Mol Sci. 2023 Jan 18;24(3):1944. doi: 10.3390/ijms24031944 (PMC9916151; doi:10.3390/ijms24031944)
Supplement: Supplementary file 1 [file ijms-24-01944-s001.zip › ijms-1991635-supplementary.pdf]

SUPPLEMENTARY FIGURES

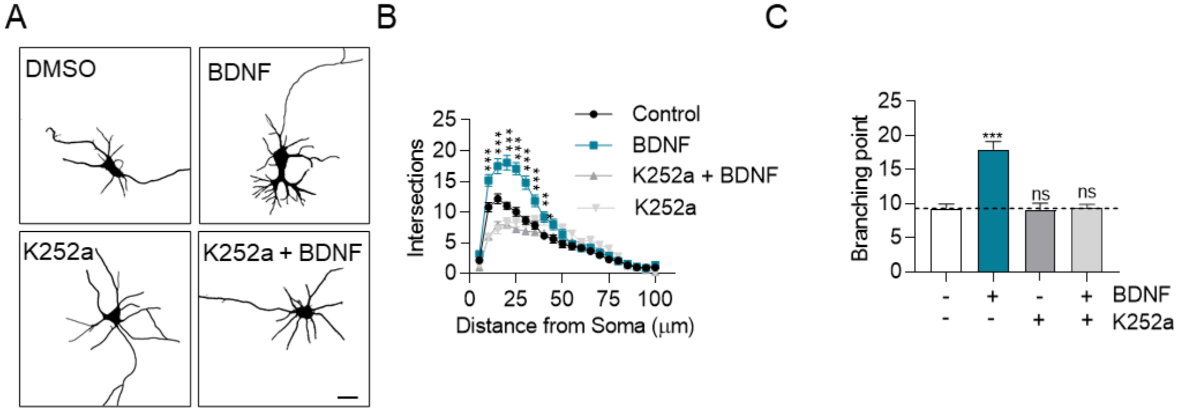

Supplementary Figure S1: Effect of Trk receptor inhibition on BDNF-induced dendritic growth. A-C) 7 DIV rat primary hippocampal neurons were stimulated for 48 hours with 50 ng/ml BDNF. The neurons were fixed and a somatodendritic compartment marker, MAP2, was immunostained. Images were captured on a Zeiss Axiovert 2000 inverted confocal microscope. A**)** Representative images of hippocampal neurons under conditions of DMSO (control), BDNF, K252a, and K252a + BDNF treatment (Scale bar, 10 μm). B**)** Sholl analysis of traced neurons. C**)** Branching point quantitation. 40-60 neurons were analyzed per experimental group from three independent experiments. *$p<0.05$, **$p<0.01$, ***$p<0.001$. Statistical analysis corresponds to a one-way ANOVA test followed by Bonferroni post-test for multiple comparisons and Two-way ANOVA with Bonferroni post-test for multiple comparisons. Results are expressed as ± SEM.

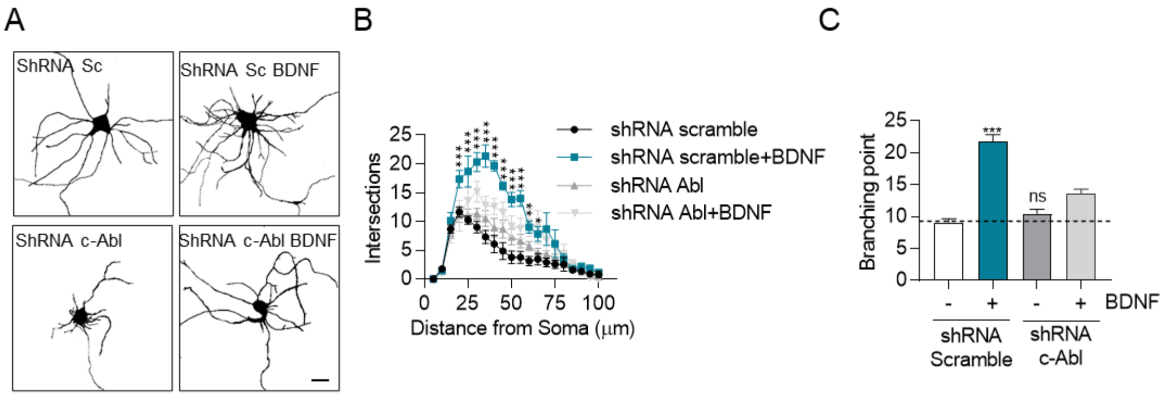

Supplementary Figure S2: Ablation of c-Abl expression by shRNA transfection prevents BDNF-induced dendritic growth in hippocampal neurons. A-C) 5 DIV rat hippocampal neurons were transfected with a scrambled shRNA or a c-Abl-targeting shRNA. After 2 days of shRNA expression, neurons were stimulated for 48 hours with 50 ng/ml BDNF. Neurons were fixed and MAP2 was immunostained to label the somatodendritic compartment. Images were captured on a Zeiss Axiovert 2000 inverted confocal microscope. A) Representative images of hippocampal neurons under conditions of shRNA scramble, shRNA scramble+BDNF, shRNA c-Abl or shRNA c-Abl+BDNF treatment (Scale bar, 10 μm). B) Sholl analysis of traced neuron C) Branching point quantitation. 40-60 neurons were analyzed per experimental group from three independent experiments. *p<0.05, **p< 0.01, ***p< 0.001. Statistical analysis corresponds to a one-way ANOVA test followed by Bonferroni post-test for multiple comparisons and Two-way ANOVA with Bonferroni post-test for multiple comparisons. Results are expressed as ± SEM.

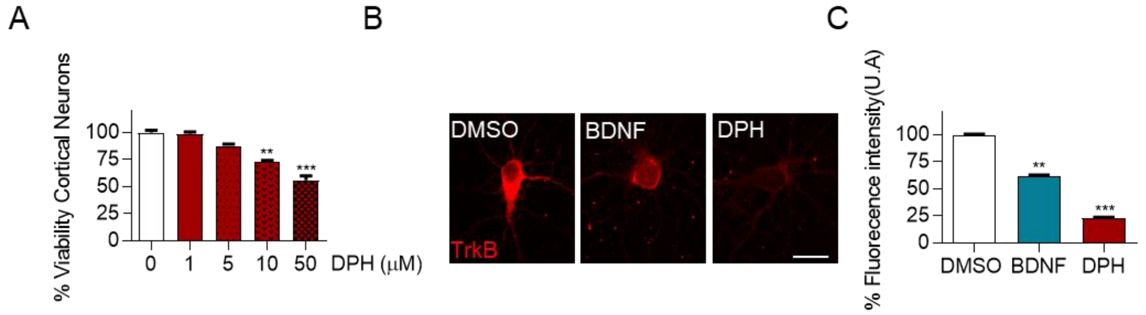

Supplementary Figure S3: Effects of DPH on cell viability and surface TrkB. A**)** Rat cortical neurons (7DIV) were stimulated with different concentrations of DPH (1, 5, 10 and 50 μM) for 24 hours. 40.000 cells were seeded per well. Cell viability was evaluated by a MTT assay. B**)** Seven DIV rat primary hippocampal neurons were treated with BDNF or DPH for 30 min, and then were fixed and immunostained with an antibody directed against an extracellular epitope of the TrkB receptor (Scale bar, 10 μm). C) Quantification of TrkB percentage fluorescence intensity. 20-30 neurons were analyzed per experimental group from three independent experiments. *p<0.05, **p< 0.01, ***p< 0.001. Results are expressed as ± SEM.

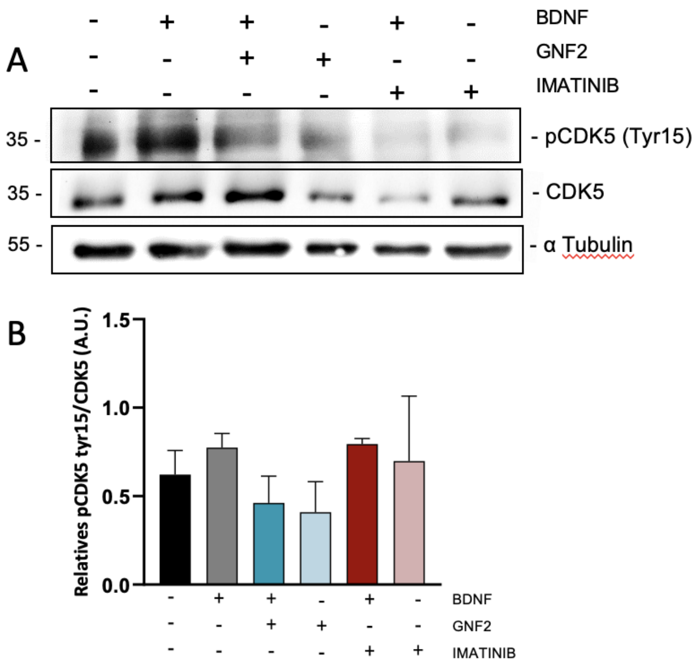

Supplementary Figure S4. Changes in phosphorylated CDK5 (Tyr-15) in response to BDNF treatment and c-Abl inhibition. **(A)** Rat hippocampal neurons were pretreated with 5 μM Imatinib or GNF2, for 1 hour, and then were stimulated with 50 ng/ml of BDNF for 1 hour. Protein extracts were immunoblotted for phospho-CDK5 (Tyr15), CDK5, and Alpha tubulin levels. **(B)** Densitometric quantification of relative phospho-CDK5/CDK5 levels. Results correspond to 2 independent experiments, and are displayed as the mean + SEM.
